# Treatment Challenges of Acquired Thrombotic Thrombocytopenic Purpura in Pediatric Patients From a Low-Income Country

**DOI:** 10.7759/cureus.45540

**Published:** 2023-09-19

**Authors:** Willy Nava Gutiérrez, Juan-Daniel Garza-Escobar, Adriana-Carolina Sandoval-González, César-Alejandro Alonso-Tellez

**Affiliations:** 1 Hematology and Oncology, Universidad de Monterrey, Monterrey, MEX; 2 Hematology and Oncology, Instituto Mexicano Del Seguro Social, Monterrey, MEX; 3 Pediatric Hematology, Instituto Mexicano Del Seguro Social, Monterrey, MEX; 4 Research, Instituto Mexicano Del Seguro Social, Monterrey, MEX

**Keywords:** low- to middle-income countries, adamts13, acute hemolytic anemia, hematology emergencies, thrombotic microangiopathy (tma), therapeutic plasma exchange (tpe)

## Abstract

This study presents a comprehensive analysis of two cases of acquired thrombotic thrombocytopenic purpura (aTTP) observed in pediatric patients from a low-income country. In the instances described, both patients underwent a treatment regimen involving plasma exchange and immunosuppressive therapy conducted without the use of caplacizumab. Caplacizumab, an approved drug for adults known for its limited availability and high cost, has exhibited efficacy in reducing response time and recurrence frequency in aTTP cases. This approach resulted in significant clinical improvement and eventual remission of symptoms in one of the cases. These cases underscore the urgent necessity for a more inclusive approach in national health programs and international treatment guidelines. Specifically, there is a call to expand the existing comprehensive treatment algorithms to accommodate countries lacking access to caplacizumab. This adaptation aims to ensure the availability of suitable and effective treatment options for aTTP patients in regions facing limited pharmaceutical accessibility.

## Introduction

Thrombotic microangiopathies (TMAs) occurring during childhood exhibit an incidence of fewer than one case per million individuals, with acquired thrombotic thrombocytopenic purpura (aTTP) being even more uncommon [1-3]. Among the affected, girls tend to be more susceptible than boys to developing aTTP, a condition marked by severe ADAMTS-13 deficiency, which is a specific form of TMA [3]. These rare TMA conditions can result in elevated mortality rates due to a lack of appropriate treatment options. Diagnostic indicators for aTTP encompass hemolysis, elevated lactate dehydrogenase (LDH) levels, diminished haptoglobin, the presence of schistocytes in peripheral blood, as well as thrombocytopenia [4]. aTTP may arise as an idiopathic condition or be associated with other factors, such as severe acute respiratory syndrome coronavirus 2 (SARS-CoV-2) infection [2-4].

The underlying pathophysiology of aTTP is closely linked to a significant functional deficiency of ADAMTS-13, leading to the accumulation of ultralong von Willebrand Factor (vWF) multimers and spontaneous formation of platelet-rich microthrombi, subsequently causing local ischemia. Among the mechanisms altering ADAMTS-13 functionality, anti-ADAMTS-13 antibodies are the most frequently encountered [5,6].

Suspicion of aTTP in pediatric cases arises when cytopenia is evident, encompassing microangiopathic hemolytic anemia and thrombocytopenia due to consumption [1]. Historically, aTTP diagnosis was established when patients manifested a pentad of fever, renal and neurological anomalies, TMA, and thrombocytopenia, although this pentad has become less prevalent in contemporary diagnosis [7-9]. At present, most patients demonstrate thrombocytopenia, TMA, and erythrocyte fragmentation in peripheral blood, coupled with negative direct Coombs testing and no deviations in coagulation times [10,11]. Initial analysis for suspected TMA should involve assessing ADAMTS-13 activity, with a marked deficiency in activity (<10%) confirming the diagnosis of thrombotic thrombocytopenic purpura (TTP). To corroborate aTTP, quantification of ADAMTS-13 inhibitor, typically of the IgG type, is deemed essential [12].

## Case presentation

First case

An 11-year-old male patient, with no significant family history, was delivered via cesarean section due to a short intergenesic period. This birth marked the second gestation, and there were no abnormalities reported during prenatal care. In July 2021, eight days before presenting to our hospital, the patient developed a sudden onset of symptoms, including oral intolerance, asthenia, adynamia, dyspnea on moderate exertion, fever, and progressive drowsiness. These alarming symptoms prompted immediate medical attention.

Physical examination upon admission revealed a mobile left inframandibular lymph node, measuring approximately 1.5 cm in size, but there were no signs of hepatosplenomegaly. Given the clinical presentation and concerns about cytopenia, the patient underwent further evaluation and diagnostic workup.

Laboratory investigations at admission indicated that the Coombs direct test was negative. The hematological assessment revealed regenerative anemia (Table 1), and a blood smear analysis detected the presence of six to eight schistocytes per field. Polymerase chain reaction (PCR) results for SARS-CoV-2 and other viral tests returned negative results, ruling out infectious etiologies.

**Table 1:** Admission laboratory test. Ab: antibodies; Act: activity; Hb: hemoglobin; IB: indirect bilirubin; LDH: lactate dehydrogenase; Ret: reticulocyte; TB: total bilirubin

Case	Hb (g/L)	Platelet (K/µL)	Ret (%)	Creatinine (mg/dL)	LDH (×10^1^ U/L)	TB/IB (mg/dL)	ADAMTS-13 act (%)	ADAMTS-13 Ab (U/L)
# 1	73	10.1	16	0.9	222.3	2.0/1.3	0	88.18
# 2	69	20.2	17	1.1	56.5	1/0.9	0	153

During his hospital stay, the patient underwent a series of plasma exchange (PEX) procedures. The treatment regimen began with a PEX calculated at 1.5 times the patient’s blood volume, followed by three additional sessions at 1.0 times the blood volume. Despite these efforts, unfortunately, there was no significant improvement in the patient’s condition. Tragically, the patient’s condition deteriorated, and he ultimately succumbed to a stroke.

Second case

A 13-year-old female, with no relevant family history, was the product of the fourth gestation with adequate prenatal care. She was born via cesarean section due to pelvic presentation. In November 2021, she presented with a sudden onset of a holocranial headache, characterized by an intensity ranging from 6 to 8 on the Visual Analog Scale. The headache was accompanied by episodes of vomiting, primarily containing gastric contents. Initially, the clinical presentation raised suspicion of appendicitis, prompting an immediate evaluation.

Upon further examination, cytopenia was identified, leading to a referral to the hematology department for comprehensive assessment. Physical examination revealed a pale appearance, along with grade I hepatomegaly, although no splenomegaly was observed.

**Figure 1:**
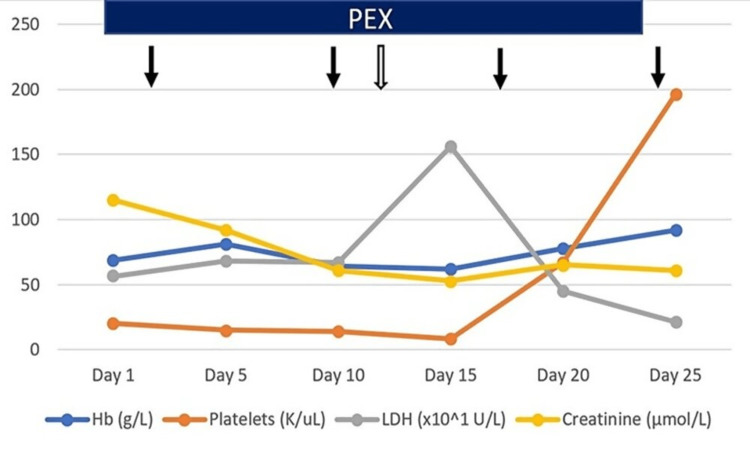
Follow-up of case two. Black arrows: rituximab. White arrow: cyclophosphamide and beginning of mycophenolate mofetil. Hb: hemoglobin; LDH: lactate dehydrogenase; PEX: therapeutic plasma exchange

Laboratory investigations (Figure 1, Table 1) yielded a PLASMIC Score of 6 points, while tests for direct Coombs, SARS-CoV-2 PCR, and other viral infections returned negative results. Given these findings, a diagnosis of aTTP was considered.

The treatment approach for this patient consisted of initiating PEX at 1.5 times her blood volume initially, followed by a reduced volume of 1.0 times her blood volume. On the third day of hospitalization, 500 mg of rituximab was administered intravenously on a weekly basis for a total of four weeks. PEX was temporarily halted for 24 hours following each rituximab infusion. Additionally, a single dose of cyclophosphamide (1,000 mg) was introduced on the 13th day of treatment due to an inadequate initial response. This was administered concurrently with a daily dose of 1,500 mg of mycophenolic acid.

By the fifth PEX session, the patient’s platelet count had exceeded 150 K/µL, and LDH levels had normalized. However, a transient ischemic attack occurred as a complication during the course of treatment. Notably, no structural abnormalities were detected in the computerized tomography scans performed.

Following her recovery from the transient ischemic attack, the patient was discharged from the hospital after receiving the third dose of rituximab on the 24th day of her hospital stay. She was prescribed mycophenolic acid for a three-month maintenance period. Currently, she remains in a surveillance phase, free from treatment, and asymptomatic.

## Discussion

aTTP is a medical emergency that necessitates immediate treatment due to its potential to cause severe harm or fatality in patients, as initially described by Moschcowitz in 1924 in a 16-year-old adolescent. Following the 1960s, the earliest reports of TTP in pediatric patients began to surface. It was not until the late 1970s that the congenital deficiency of ADAMTS-13 was designated as Upshaw-Schulman syndrome [11,12].

The most common cause of TTP in the pediatric age group is congenital ADAMTS-13 deficiency, in contrast to aTTP, which is less prevalent. The primary approach for aTTP treatment involves performing PEX utilizing fresh frozen plasma and steroids, which is recognized as the initial therapeutic choice. A delayed commencement of plasma exchange has shown an association with a heightened early mortality rate, potentially stemming from misdiagnosis or insufficient access to treatment, similar to what was observed in our patient [11-13].

Alongside PEX, caplacizumab presents a novel treatment avenue for adult aTTP patients by obstructing the A1 domain of vWF. However, this medication remains unapproved for pediatric usage [13]. Recently, dosing proposals have emerged through pharmacological models, suggesting a dosage of 5 mg for children weighing less than 40 kg and 10 mg for those over 40 kg and/or 12 years of age [14]. An additional prospective therapeutic option for pediatric aTTP is N-acetylcysteine, which regulates vWF polymerization and adhesion, yet necessitates further investigation [1].

Immunosuppressive therapy additionally plays a role in mitigating the production of anti-ADAMTS-13 antibodies. The employment of anti-CD20 (rituximab) in combination with PEX has exhibited favorable outcomes. Other immunosuppressive agents such as cyclosporin A, mycophenolate mofetil, or cyclophosphamide may offer potential benefits in refractory cases, which are characterized as situations where remission is not attained by the fifth day, similar to what was observed in the second case [11-13,15,16]. Supportive therapy also stands as a crucial component for aTTP pediatric patients, encompassing the administration of blood components and folic acid during hemolysis [13,15-19].

## Conclusions

In the landscape of TMAs, aTTP emerges as a rare subtype, particularly among children. The significance of timely diagnosis and prompt initiation of plasma exchange cannot be overstated, as these factors profoundly influence the ultimate outcome of the disease. With the advent of caplacizumab, the occurrence of refractory disease in adults has notably diminished, predominantly in developed nations. Consequently, this progress has led to the exclusion of immunosuppressive agents such as cyclophosphamide and mycophenolate mofetil from the therapeutic repertoire outlined in the guidelines of the International Society of Thrombosis and Hemostasis. Regrettably, this exclusion leaves developing countries with limited access to these advancements. Furthermore, there is still insufficient evidence for the use of this drug in the pediatric population, prompting the use of pharmacological models to benefit this group and the reporting of cases like this one to generate valuable evidence for daily clinical practice.

In light of this pressing disparity, we earnestly advocate for guideline reviewers to recognize and address this real-world scenario in the pediatric population. It is imperative that future updates to the guidelines be adapted to the global context, acknowledging the limitations faced by healthcare systems with restricted access to advanced treatments. The urgent need for comprehensive, equitable, and inclusive treatment approaches for aTTP pediatric patients across diverse healthcare settings cannot be overstressed. By embracing this reality and tailoring recommendations accordingly, we aspire to enhance the care and outcomes of aTTP pediatric patients worldwide.
